# Condition-Adaptive CNN with Spatiotemporal Fusion for Enhanced Motor Fault Diagnosis

**DOI:** 10.3390/s26041314

**Published:** 2026-02-18

**Authors:** Jin Lv, Lixin Wei, Yu Feng

**Affiliations:** 1 School of Electrical Engineering, Yanshan University, Qinhuangdao 066004, China; lvkim@163.com (J.L.); wlx2000@ysu.edu.cn (L.W.); 2 Tianjin Electrical Science Research Institute Co., Ltd., Tianjin 300180, China

**Keywords:** motor fault diagnosis, convolutional neural network, self-attention mechanism, swarm intelligence optimization, industrial condition monitoring

## Abstract

Electric motors are widely used in industrial production systems, and various fault modes may occur during long-term operation under complex and noisy conditions. Accurate fault diagnosis remains challenging, especially when signal characteristics vary depending on the operating state. To address this issue, this paper presents a fault diagnosis framework based on a convolutional neural network (CNN), which features adaptive parameter optimization and enhanced feature representation. This method integrates the bee colony algorithm (BCA) into CNN training, adaptively adjusts the model parameters based on signal conditions, and shortens the convergence time compared to traditional gradient-based optimization. In order to improve the extraction of high-frequency and transient fault features, a spatiotemporal fusion architecture is designed, which combines large-kernel convolution, a bottleneck layer, and an improved self-attention (ISA) mechanism. In addition, an engineering-oriented data augmentation strategy based on multi-scale window offset and noise superposition has been applied to one-dimensional vibration signals to improve the robustness of the model. The proposed CNN-BCA-ISA framework is evaluated using a mixed dataset consisting of on-site data collected from a steel plant and a public dataset from Case Western Reserve University (CWRU). The experimental results show that the diagnostic accuracy is 96.4%, and the performance is stable under different noise levels, indicating good generalization abilities under various operating conditions. In addition, a real-time fault diagnosis system based on the proposed framework has been implemented and validated in industrial environments, confirming its feasibility in practical state monitoring applications.

## 1. Introduction

With the rapid development of modern industrialization, electric motors have been widely adopted across various industrial sectors. However, prolonged operation, harsh field environments, and human factors contribute to frequent motor faults, requiring labor-intensive manual troubleshooting that is time-consuming and costly [1]. Traditional fault diagnosis methods primarily rely on expert experience and on-site operator analysis, exhibiting low efficiency, poor reliability, and high operational costs [2].

### 1.1. Literature Review on Intelligent Fault Diagnosis Methods

#### 1.1.1. Deep Learning-Based Approaches

Convolutional neural networks (CNNs) have become dominant in fault diagnosis due to their strong feature extraction capabilities. Wang et al. [3] demonstrated excellent performance using CNNs in laboratory settings, while Xie et al. [4] introduced continuous wavelet transforms and TransXNet with multi-source information fusion via test rig simulations. Yin et al. [5] developed a deep residual network approach using multi-sensor data processed through variational mode decomposition and a short-time Fourier transform for nuclear pump motor fault recognition. However, standard CNNs often suffer from fixed architectures that lack adaptability to varying operational conditions.

#### 1.1.2. Hybrid Models Integrating Signal Processing and Deep Learning

To improve fault feature extraction from non-stationary vibration signals, several studies have explored the integration of classical signal processing techniques with deep learning models. Song et al. [6] combined variational mode decomposition with deep neural networks and demonstrated its effectiveness for bearing fault classification. Huang et al. [7] employed recursive analysis together with ensemble learning methods to enhance the fault identification performance.

Although these hybrid approaches improve feature representation by incorporating domain knowledge, they typically rely on manually designed preprocessing steps or handcrafted decomposition strategies. As a result, the overall diagnostic performance remains sensitive to parameter selection, and true end-to-end optimization is often not achieved.

#### 1.1.3. Optimization-Enhanced Deep Learning Frameworks

Another research direction focuses on improving the adaptability of deep learning models through optimization techniques. Ren et al. [8] enhanced activation functions to achieve rapid convergence and high diagnostic accuracy without explicit manual feature extraction. In addition, swarm intelligence algorithms such as particle swarm optimization (PSO) [9], genetic algorithms (GAs), and ant colony optimization (ACO) have been introduced for hyperparameter tuning in fault diagnosis networks.

While these studies demonstrate the potential of optimization-assisted learning, most existing methods treat parameter optimization and feature extraction as separate problems. Consequently, the resulting frameworks often lack coordinated adaptation when the operating conditions vary, which limits their applicability in complex industrial environments.

#### 1.1.4. Large Language Models for Industrial Intelligence

Recent research has also explored large language models (LLMs) and visual language models (VLMs) for industrial fault diagnosis and intelligent detection, utilizing multimodal learning and instruction alignment to address challenging robustness issues such as data imbalance. For example, CNC-VLM uses an RLHF-optimized industrial visual language model for unbalanced CNC fault detection, demonstrating the potential of the model in industrial scenarios. Unlike the VLM-based paradigm, which mainly focuses on semantic/multimodal understanding, this paper focuses on signal spatiotemporal feature learning with parameter adaptation (BCA) and real-time deployment constraints under actual working conditions and strong noise. Extending deep learning models and large-scale modeling ideas to time series vibration diagnosis (such as the multimodal alignment of sensor signals) is a promising research direction [10].

### 1.2. Research Gaps and Challenges

Despite continuous progress, several fundamental challenges remain unresolved in current motor fault diagnosis systems.

First, standard convolutional neural networks with fixed kernel sizes have limited capabilities to simultaneously capture local transient characteristics and long-term operational trends in time series signals, leading to fragmented feature representations [11].

Second, commonly used fixed-architecture networks, such as the Visual Geometry Group (VGG) and Residual Network (ResNet), exhibit limited adaptability to changing load conditions and operational states. This parameter rigidity often results in degraded diagnostic performance under non-stationary industrial conditions [12].

Third, real-world industrial environments introduce various noise sources, including electrical interference, mechanical vibration, and sensor noise. These disturbances significantly affect diagnostic robustness, with reported reliability reductions ranging from 15% to 40% in practical deployments [13,14].

Although several studies have incorporated swarm intelligence algorithms or attention mechanisms into CNN-based models [15,16,17,18], most of them focus on isolated improvements in either parameter optimization or temporal modeling. A unified spatiotemporal fusion framework that can adapt to dynamic operating conditions while maintaining robustness against environmental disturbances remains insufficiently explored.

The existing research on fault diagnosis based on CNNs mainly develops along two paths: (i) introducing attention mechanisms to enhance feature selection and long-range dependency modeling; (ii) introducing swarm intelligence/metaheuristic algorithms to reduce the manual tuning costs or improve the global search capabilities. However, most of the work only focuses on isolated improvements in one aspect mentioned above, and there are still three shortcomings:

(1) Insufficient adaptation to changing operating conditions:The attention module typically operates under a fixed network structure and fixed hyperparameter configuration. When faced with changes in speed/load, feature distribution drift can cause the optimal hyperparameter configuration to vary with changing operating conditions.
(2) Insufficient robustness under coupling of strong noise and variable operating conditions:It is difficult to balance noise suppression and cross operating condition generalization solely through attention or hyperparameter searching.
(3) Insufficient engineering closed-loop verification:Many methods remain at the level of public datasets or offline evaluations, lacking real-time system deployability verification.

To this end, this article proposes a conditional adaptive CNN framework: using BCA to perform a global search in a mixed discrete continuous hyperparameter space to achieve the adaptive configuration of operating conditions and using improved self-attention (ISA) to achieve spatiotemporal joint modeling, thereby improving the diagnostic performance and engineering usability under the combined context of variable operating conditions and strong noise.

### 1.3. Contributions and Novelty

Inspired by the above challenges, this study proposes an integrated motor fault diagnosis framework, which contributes as follows.

Methodological contributions:

(1) We propose a conditional adaptive hyperparameter optimization strategy: in variable operating conditions, we perform a global search and the adaptive configuration of key hyperparameters through BCA to reduce the dependence on manual parameter tuning and improve the convergence stability.
(2) We propose a spatiotemporal fusion structure that unifies the local pattern extraction of large-kernel convolution and the global dependency modeling of ISA into the same framework to enhance the robust representation of noise disturbances and cross-condition distribution drift.

Engineering contributions:

(3) We conduct real-time deployment verification for industrial sites, achieve end-to-end data collection and enhancement, and verify availability and stability under actual working conditions and noise backgrounds.

### 1.4. Paper Organization

The remainder of this paper is organized as follows. Section 2 introduces the construction of the integrated dataset and data fusion procedure. Section 3 describes the proposed CNN-BCA-ISA fault diagnosis framework in detail. Section 4 presents the experimental setup and validation results. Finally, Section 5 summarizes the main conclusions and outlines future research directions.

**Figure 1:**
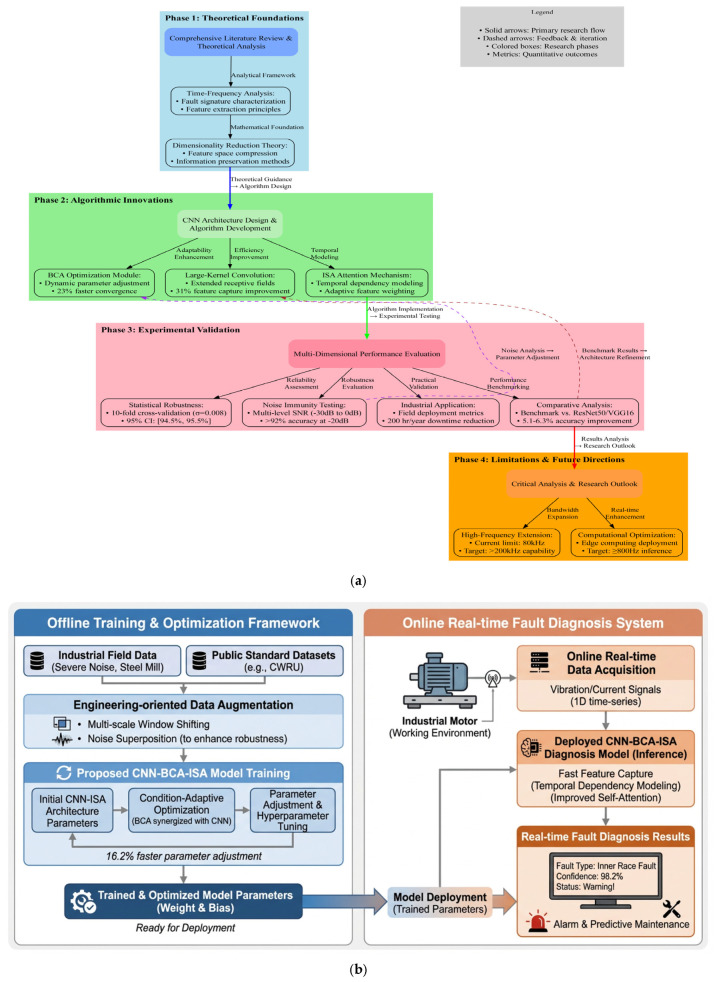
(**a**) Overall flowchart of the proposed fault diagnosis framework; (**b**) system architecture diagram showing offline training and online recognition stages.

The overall framework of the proposed motor fault diagnosis method is as follows (Figure 1a). The framework illustrates the relationship between theoretical analysis, algorithm design, and engineering validation. Theoretical studies on motor fault mechanisms and signal characteristics provide guidance for model development. Based on these insights, the CNN-BCA-ISA architecture is constructed by integrating adaptive parameter optimization, large-kernel convolution, and an improved self-attention mechanism. The proposed method is evaluated through cross-validation, noise robustness tests, and industrial application scenarios. Experimental observations and practical limitations are further used to inform model refinement and future research.

The system architecture diagram showing the offline training and online recognition stages is shown in Figure 1b.

## 2. Construction of Integrated Dataset

### 2.1. Field Data from Industrial Project

In an overseas steel continuous rolling mill project undertaken by the authors’ company, dedicated communication devices were deployed on-site to acquire operational data from motors during production. Utilizing the M107 vibration sensor (sensitivity: 100 mV/g) installed on the motor bearing housing and the LEMIT 200S current sensor for electrical signals, data were streamed via a Siemens SL150 controller to the Industrial Big Analysis Platform for Data Acquisition (IBAPDA) at a 10 kHz sampling rate. The established communication infrastructure captured multi-parametric signals with a 1 ms sampling period, including

1. Current and voltage profiles;
2. Rotational speed measurements;
3. Triaxial vibration signatures.

The dataset encompasses five asynchronous motors (Siemens 1LE0 series) with power ratings of 4000–7000 kW, operating under varying loads (30–100% of rated capacity) over 18 months. Controlled fault data were acquired during scheduled maintenance windows. For instance, bearing faults were simulated by introducing single-point defects (0.2–0.5 mm depth) on inner/outer races via electric engraving, while stator inter-turn shorts were emulated using tapped windings with programmable contactors to create precise short-circuit ratios (5–20%). For inter-turn short-circuit (ITSC) faults, the severity was defined by the proportion of shorted turns relative to the total turns of the affected winding. Three discrete severity levels were considered in this work, 5%, 10%, and 20%, which were implemented to ensure repeatable experiments and consistent labeling.

The vibration signals used in this study were collected on an industrial motor test platform deployed in a steel plant environment. To ensure reliable labeling and controllable fault severity, representative motor faults were introduced in a controlled and repeatable manner on the test motor while maintaining the same on-site operating environment. This design allowed the dataset to capture realistic industrial noise and operating disturbances while providing accurate annotations for the fault type and severity.

**Figure 2:**
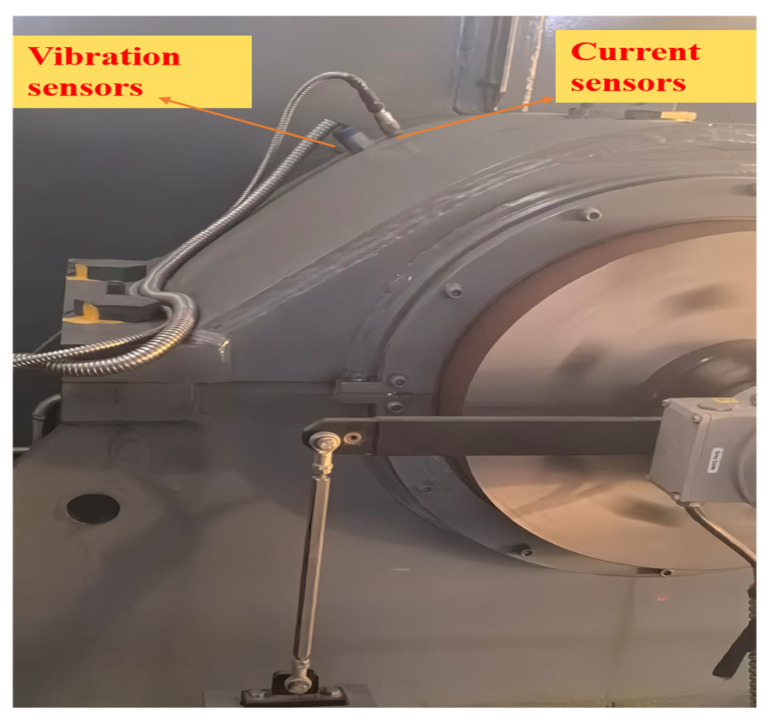
Sensors from industrial project.

In addition to faulty conditions, vibration signals under normal operating conditions were collected as healthy samples. Each record was annotated with the operating state (healthy/faulty), fault type, fault location, severity level, and load condition. To improve the class balance, stratified sampling was adopted, and minority fault categories were augmented via oversampling, ensuring that the training set contained sufficient examples for each fault class. The specifications for the on-site data collection system are shown in Table 1, and the placement of on-site sensors is shown in Figure 2.

### 2.2. CWRU Bearing Dataset

The Case Western Reserve University (CWRU) Bearing Data Center’s publicly available dataset was incorporated; this is widely recognized for its reliability and representativeness in bearing fault diagnosis research. This dataset can be accessed at the following: https://engineering.case.edu/bearingdatacenter/download-data-file (accessed on 15 January 2026). This benchmark dataset contains vibration signals covering

1. Multiple fault types (inner race, outer race, ball defects);
2. Variable load conditions (0–3 hp);
3. Different damage severities (0.007–0.028-inch diameters).

The CWRU dataset provides a standardized benchmark for comparing fault diagnosis algorithms and has been widely used in the research community.

### 2.3. Dataset Integration and Fault Taxonomy

The field-collected data and CWRU dataset were systematically integrated through the following.

**Figure 3:**
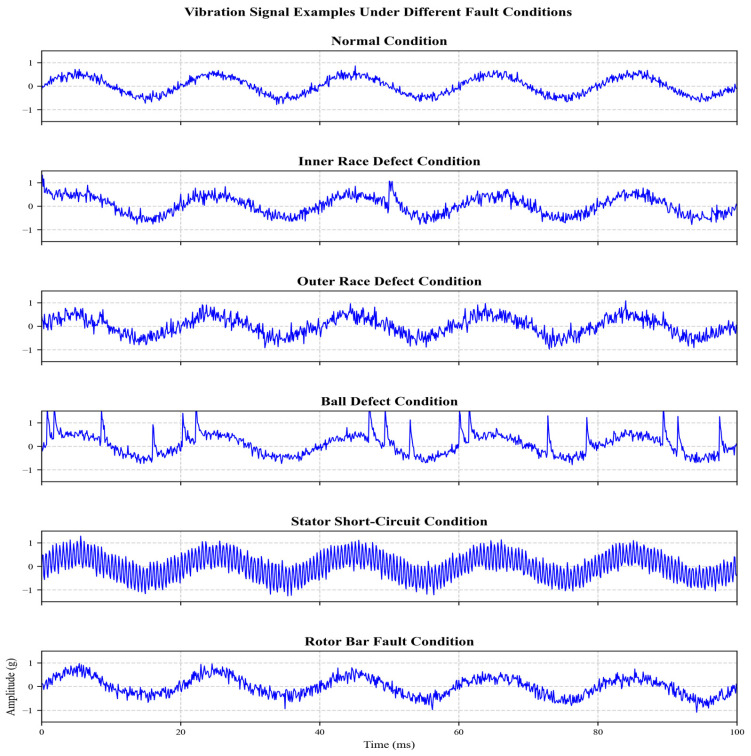
Signal examples from different fault conditions.

1. Data format standardization—unified time series storage format (HDF5).
2. Label consistency alignment:

Normal/abnormal state classification;

Fault type taxonomy harmonization;

Load condition mapping. (Because the CWRU dataset reports operating conditions using mechanical load indices in horsepower (hp), while the field dataset used the percentage of rated load, we unified both into normalized load categories (low/high) for cross-dataset fusion. In particular, 0 hp in CWRU is mapped to a low load and values of 1–3 hp are mapped to high loads to align with the two rated load levels used in the field acquisition.) Examples of signals from different fault conditions are shown in Figure 3.

3. Operational context annotation:

Tagging field-specific working cycles;

Documenting environmental variables (temperature, humidity).

To address class imbalance, this study applied stratified sampling and oversampling for minority fault categories. The resulting integrated dataset’s composition is shown in Table 2. The types of faults caused by natural operation and artificially induced faults are shown in Table 3 (due to the low occurrence of faults caused by natural operation, they were artificially induced).

### 2.4. Data Fusion Procedure

**Figure 4:**
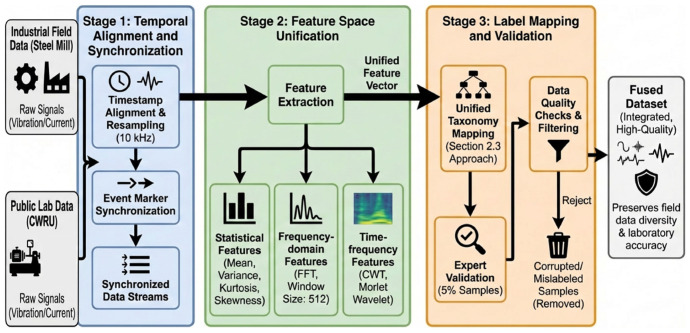
Data fusion process.

Figure 4 illustrates the data fusion process, which involved three main stages.

Stage 1: Temporal Alignment and Synchronization

Raw signals from different sources are aligned using timestamps and resampled to a common sampling rate (10 kHz).

Event markers from the steel mill data are synchronized with CWRU fault injection events.

Stage 2: Feature Space Unification

Statistical features (mean, variance, kurtosis, skewness) are extracted from both datasets.

Frequency-domain features are computed using the FFT with consistent window sizes (512 samples).

Time–frequency features are extracted using the continuous wavelet transform with Morlet wavelets.

Stage 3: Label Mapping and Validation

Fault labels are mapped to a unified taxonomy (as described in Section 2.3).

Expert validation is performed on 5% of the samples to ensure labeling accuracy.

Data quality checks remove corrupted or mislabeled samples.

The fused dataset preserves the advantages of both sources: the diversity and realism of field data and the controlled conditions and ground truth accuracy of laboratory data.

## 3. CNN-BCA-ISA-Based Fault Diagnosis Model

**Figure 5:**
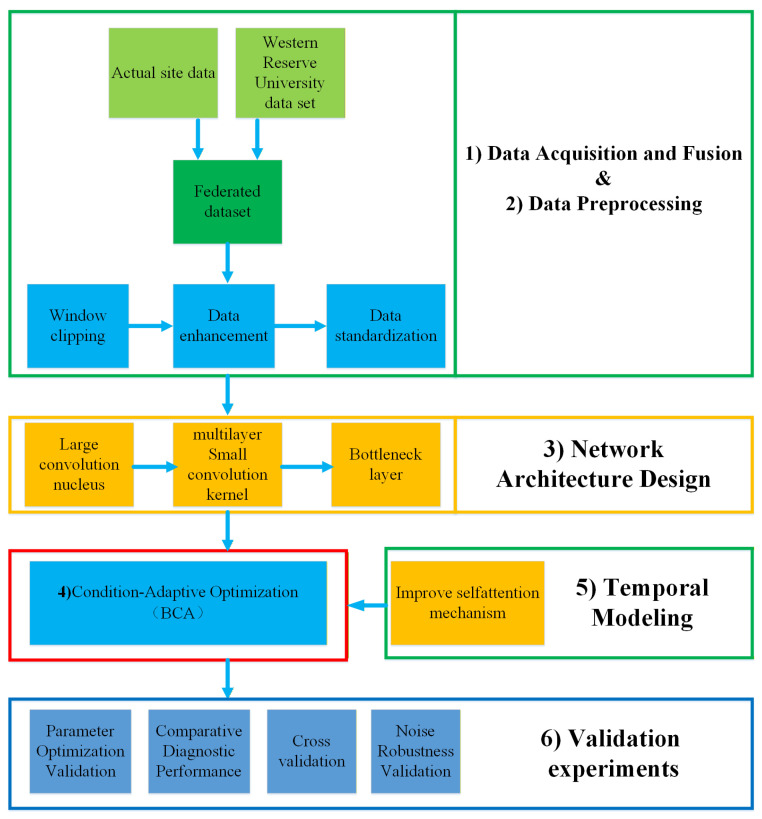
Technical roadmap of this work.

The field investigations and literature review reveal that bearing-related faults constitute approximately 40% of motor failures, while stator current short-circuit faults account for about 30%. Consequently, accurate diagnosis of these two predominant failure types is critical for ensuring production line stability [19,20,21]. This study employs the established integrated dataset with the technical workflow illustrated in Figure 5.

(1) Data Acquisition and Fusion: Multi-source data collection, preprocessing, and fusion as described in Section 2.
(2) Data Preprocessing: Signal normalization, noise filtering, window segmentation, and data augmentation.
(3) Network Architecture Design: CNN with large-kernel convolutions, bottleneck layers, and ISA mechanism.
(4) Condition-Adaptive Optimization: BCA-based hyperparameter optimization with fitness evaluation.
(5) Temporal Modeling: ISA mechanism for capturing long-range dependencies.
(6) Validation experiments: The validation and comparative experiments indicated in the roadmap (Figure 5) are detailed in Section 4.

### 3.1. Data Preprocessing

The performance of neural network-based fault diagnosis models is strongly influenced by the quality and diversity of the input data. In industrial monitoring scenarios, vibration signals are typically collected as long one-dimensional time series, which require segmentation and normalization before being used for model training. Since common image augmentation techniques are not directly applicable to these data, a window-based preprocessing strategy is adopted in this study.

Window Cropping:

Define raw sequence x = {x_1_,x_2_,……,x_N_}, with length *N*. Set window size *W* = 512 and stride *s* = 20. Generate subsequences:

(1) $$X_{i}=\{X_{i},X_{i+1},\ldots \ldots ,X_{i+w-1}\}\mathrm{for}I=0,s,2s,\ldots \ldots ,[\frac{N-W}{S}]\cdot s$$

**Figure 6:**
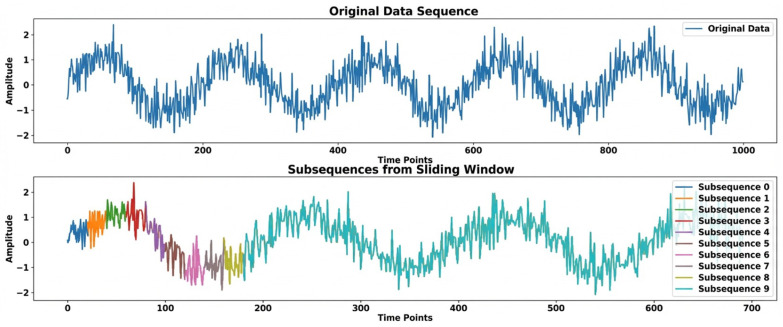
Window cropping (size = 512, stride = 20).

This operation produces a set of subsequences $\left\{x_{0},x_{s},x_{2s},\ldots \right\}$, each preserving local temporal continuity while significantly reducing the length of individual inputs. Compared with using long signals, this strategy lowers the computational cost and facilitates the learning of localized fault-related patterns. The window cropping process is illustrated in Figure 6.

Data Augmentation:

In order to further enhance the robustness of the model under variable operating conditions, a data augmentation strategy tailored to one-dimensional time series signals was adopted. Firstly, by changing the window length (e.g., 256, 512, and 1024 samples) and step size to apply multi-scale window offset, the model can observe fault features at different temporal resolutions.

Secondly, we add the standard deviation at 0.01 times the root mean square (RMS) of the original signal. This step aims to simulate common sensor noise and electromagnetic interference in industrial environments. By increasing the diversity of the training samples, the combination enhancement strategy improves the model’s generalizability to unknown operating conditions.

Data Standardization:

Industrial vibration data often contain amplitude variations, baseline drift, and occasional outliers, which can negatively affect model training. To alleviate these effects, each subsequence $X_{i}$ is standardized as follows:

(2) $$X_{i}^{\prime }=\frac{X_{i}-\mu }{\sigma }$$

where μ and σ denote the mean and standard deviation of $X_{i}$. This normalization process centers the data around a zero mean and scales them to unit variance, reducing the influence of amplitude-related variations that are not directly associated with fault dynamics. As a result, the network becomes less sensitive to irrelevant signal fluctuations, leading to more stable training and improved diagnostic performance [22].

### 3.2. Convolutional Neural Network Architecture

Convolutional neural networks aim to balance feature extraction capabilities and computational efficiency. In the first convolutional layer, a larger one-dimensional kernel of 15 × 1 is used to capture broader contextual information from the input signal. The extended receptive field enables the network to recognize complex fault-related patterns across multiple sampling points [23].

However, stacking large-kernel convolutions throughout the entire network will greatly increase the computational cost and the risk of overfitting. To address this issue, the subsequent five convolutional layers adopt smaller kernels of 5 × 1. When arranged in sequence, these smaller kernels can approximate the effects of larger receptive fields while requiring fewer floating-point operations. Compared to configurations that only use a large kernel, the hybrid kernel design reduces the computational complexity by approximately 42% based on floating-point counting [24]. The calculation assumes typical feature map dimensions and channel counts, using the standard *FLOPs* formula for a 1D convolution:

(3) $$FLOPs=2\times C_{in}\times C_{out}\times K\times L_{out}$$

where $C_{in}$ and $C_{out}\;$ are the input/output channels, K is the kernel size, and $L_{out}$ is the output length. The hybrid design reduces the dominant K-terms in five out of six layers, significantly reducing the computational complexity.

The core convolutional operation can be expressed as

(4) $$S\left(i,j\right)=\underset{m}{\sum }\underset{n}{\sum }I\left(i+m,j+n\right)\cdot K\left(m,n\right)$$

where

*I*: zero-padded input tensor;
*K*: convolution kernel weights;
*S*: output feature map;
(*i*, *j*): spatial coordinates in output;
(*m*, *n*): kernel coordinates.

A ReLU activation function follows each convolution to introduce non-linearity:

(5) $$f\left(x\right)=max\left(0,x\right)$$

This widely adopted activation mitigates gradient vanishing issues and enables non-linear function approximation.

Bottleneck layers are incorporated to reduce the parameters while preserving accuracy. Channel reduction: 1 × 1 convolution compresses the input channels. Feature learning: 3 × 1 convolution extracts spatial features. Channel expansion: 1 × 1 convolution restores the channel dimension. Each layer includes batch normalization (BN) and ReLU activation, accelerating convergence by 37% (specifically, the 37% convergence acceleration is observed when comparing the training epochs needed to achieve a loss of 0.05 with the case without bottleneck layers) [25] and improving generalization.

**Figure 7:**
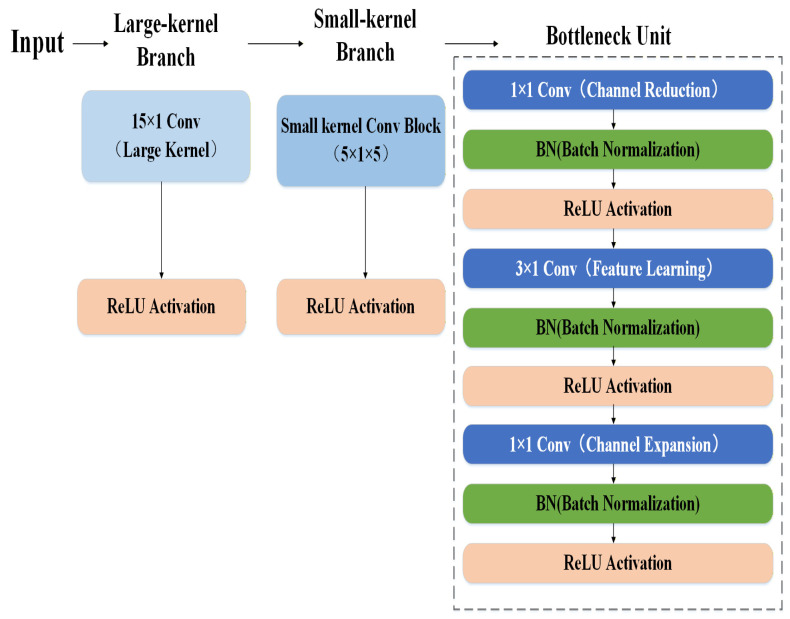
Detailed block structure.

The proposed network adopts a sequential convolutional backbone network, which is composed of large convolution kernels and multiple small convolution kernels. Then, a bottleneck layer is introduced, and the feature map is gradually extracted and refined along the depth of the network. The detailed block structure is visualized in Figure 7.

### 3.3. BCA-Based Hyperparameter Optimization

#### 3.3.1. Algorithm Selection Rationale

The selection of the bee colony algorithm (BCA) over other metaheuristics was driven by its superior balance between exploration and exploitation, which is critical for optimizing the high-dimensional, non-convex loss landscapes of convolutional neural networks (CNNs). Unlike gradient-based methods prone to local minima, population-based algorithms like BCA, particle swarm optimization (PSO), and genetic algorithms (GAs) are more suitable. However, PSO performs less well than BCA in terms of suitability for CNNs, and the convergence speed of the GA is very slow. Table 4 presents a comprehensive comparison of BCA with particle swarm optimization (PSO), the genetic algorithm (GA), differential evolution (DE), and ant colony optimization (ACO) for CNN hyperparameter optimization.

BCA alleviates these issues through its tripartite foraging: worker bees conduct a local search, follower bees bias their search towards promising areas, and scout bees avoid stagnation by reintroducing randomness. This structure is particularly effective in the hyperparameter optimization of CNNs, as the parameters (such as the convolutional kernel size and layer depth) are often interdependent. The comparative analysis summarized in Table 4 confirms the advantages of BCA in terms of convergence speed and robustness.

The evaluation criteria are quantified as follows.

Convergence Speed: Average iterations to reach 99% of the final best accuracy (fast: <100; medium: 100–300; slow: >300).

Global Search Ability: Percentage of independent runs (out of 30) where the algorithm finds a solution within the top 1% of the global optimum (good: 85–90%; very good: 90–95%; excellent: >95%).

Parameter Sensitivity: Standard deviation of final accuracy across runs with default parameter variations of ±20% (low: <0.5%; medium: 0.5–1.5%; high: >1.5%).

Computational Cost: Normalized runtime per iteration relative to the simplest algorithm (basic PSO set as 1.0).

Suitability for CNNs: A composite score (1–5) based on the weighted performance across the above four criteria.

Due to the complex structure of the search space, the hyperparameter optimization of convolutional neural networks is a challenging task. The typical features of optimization are multiple local optima and some non-differentiable regions, which limit the effectiveness of traditional gradient-based methods. In addition, strong parameter coupling often causes small changes, leading to significant performance changes and making stable convergence difficult.

The population-based metaheuristic algorithm provides an alternative solution by performing a global search without relying on gradient information. Among them, the bee colony algorithm (BCA) has attracted attention due to its simple control structure and adaptive search behavior. BCA simulates the foraging process of bees through three types of agents (hired bees, bystander bees, and scout bees), which collectively achieve local development and the full exploration of the search space. This collaborative mechanism allows the algorithm to evade local optima through memory-based selection while preserving promising solutions.

Compared with other swarm intelligence methods, such as particle swarm optimization and genetic algorithms, BCA typically requires fewer control parameters, which reduces the sensitivity to manual adjustments [26]. Previous studies have also reported the relatively fast convergence behavior of BCA in comparable optimization problems [27]. In addition, the independent search behavior of individual bees makes the algorithm suitable for parallel implementation, which is beneficial for computationally intensive neural network optimization tasks.

#### 3.3.2. Algorithm Process and Parameters

The selection of the hyperparameters directly affects the diagnostic performance and stability of deep learning-based fault diagnosis models. In this study, the bee colony algorithm was used to optimize key CNN parameters by simulating the foraging behavior observed in natural bee colonies. This algorithm iteratively executes candidate solution generation, information sharing, and solution updates through interactions between hired bees, bystander bees, and scout bees.

During the optimization process, hired bees explore the neighborhood of existing candidate solutions and evaluate their fitness, while bystander bees probabilistically select promising solutions based on shared fitness information. When stagnation occurs, reconnaissance bees are activated, allowing them to randomly explore new areas in the search space while maintaining population diversity. Through these mechanisms, BCA (Algorithm 1: BCA-Based Hyperparameter Optimization for CNN) achieves a balance between developing high-quality solutions and exploring untapped space.
**Algorithm 1:** BCA-Based Hyperparameter Optimization for CNN  Input:D: Training datasetM: CNN model structureBCA_params: Algorithm parameters (bee_count, max_iterations, limit_T)  Output:θ_best: Optimized hyperparameters (learning_rate, kernel_sizes, layer_depths)W_best: Optimized weightsB_best: Optimized biases  1: Initialize bee population: scouts, employed bees, onlookers  2: Encode each bee as θ_i = (W_i, B_i)  3: Define fitness f(θ_i) = CrossEntropyLoss(M(θ_i), D)  4: for iteration = 1 to MaxIterations do  5://Employed bee phase  6: for each employed bee i do  7: Generate new candidate: θ_new,i = θ_i + φ_i · (θ_i − θ_k)  8: if f(θ_new,i) < f(θ_i) then θ_i ← θ_new,i  9: end for  10://Onlooker bee phase  11: Calculate selection probability P_i = f(θ_i)/Σ f(θ_j)  12: for each onlooker do  13: Select bee i with probability P_i  14: Perform local search as in Equation (5)  15: end for  16://Scout bee phase  17: for each solution unchanged for T iterations do  18: Replace with random new solution  19: end for  20://Elite preservation  21: Update global best if improved  22: end for  23: return W_best, B_best

**BCA Parameter Settings**

Number of bees: 100 (20 scouts, 50 employed bees, 30 onlookers)

Maximum iterations: 100

Limit parameter T: 10

Learning rate: $\left[10^{-4},10^{-2}\right]$ When the change in the loss function remains below 1 × 10, the optimization process terminates for ^−5^ ten consecutive iterations.

**Optimization Workflow**

1. Initialization:

Initialize the bee population, including scout, employed, and onlooker bees, where each bee corresponds to a candidate solution vector *θ*_i_ = (*W_i_*, *B_i_*) representing the network weights and bias parameters.

2. Fitness Evaluation:

Define cross-entropy loss as fitness function *f*(*θi*).

3. Employed Bee Phase:

Each employed bee searches near the current solution:

(6) $$\begin{array}{c} W_{new,i}=W_{i}+\emptyset_{i}\left(W_{i}-W_{k}\right)\\ B_{new,i}=B_{i}+\emptyset_{i}\left(B_{i}-B_{k}\right) \end{array}$$

where $\emptyset_{i}$ ∈ [−1,1] is a random variable, and (*W*_*k*_,*B*_*k*_) is a randomly selected solution.

4. Onlooker Bee Phase:

Onlookers select solutions probabilistically

(7) $$P_{i}=\frac{f\left(W_{i},B_{i}\right)}{\sum_{j=1}^{N}f\left(W_{j},B_{j}\right)}$$

and then perform a local search using Equation (5).

5. Scout Bee Phase:

Solutions unchanged after *T* iterations are abandoned, replaced by new random solutions.

6. Elite Preservation:

Update the global best solution:

(8) $$\begin{array}{c} if\;f\left(W_{new,i},B_{new,i}\right)>f\left(W_{best},B_{best}\right)\\ W_{best}=W_{new,i}\\ B_{best}=B_{new,i} \end{array}$$

Compared to typical stochastic (e.g., random search) and gradient-based hyperparameter optimization methods, BCA demonstrates superior global search capabilities and robustness for complex CNN optimization. In field tests under a consistent evaluation protocol (terminating upon reaching target validation accuracy of 98.5%), BCA reduced the average parameter tuning time by 63% relative to these baseline approaches.

### 3.4. Enhanced Self-Attention Mechanism

#### 3.4.1. Standard Self-Attention

Fault diagnosis requires the analysis of contextual dependencies across extended time sequences. While CNNs struggle with long-range dependencies due to limited receptive fields, self-attention mechanisms excel in capturing the global context. This study integrates an improved self-attention (ISA) module to augment the temporal modeling capabilities.

For input sequence *X*∈*R*^*Ld*^ (*L*: sequence length, *d*: feature dimension):

Compute query/key/value vectors:

(9) $$\begin{array}{c} Q=XW^{Q}\\ K=XW^{K}\\ V=XW^{V} \end{array}$$

where *W*^*Q*^, *W*^*K*^, *W*^*V*^ ∈ *R*^*d*^ are projection matrices. *W*^*Q*^, *W*^*K*^, *W*^*V*^ are learnable projection matrices initialized randomly and optimized during training through gradient descent [28].

Calculate attention scores:

(10) $$Scores=softmax\left(\frac{QK^{T}}{\sqrt{d_{k}}}\right)$$

Generate contextual representation:

(11) Z=Scores·V

#### 3.4.2. Proposed Improvements

Based on the above basic principles, in order to dynamically weight key features in different contexts, the ISA module adopts the following weighting scheme.

1. Feature-Level Weighting:

(12) $$\begin{array}{c} Q=\left(X⊙W^{Q}\right)W^{Q}\\ K=\left(X⊙W^{K}\right)W^{K}\\ V=\left(X⊙W^{V}\right)W^{V} \end{array}$$

where ⊙ denotes element-wise multiplication, and *W*^*Q*^, *W*^*K*^, *W*^*V*^∈*R*^*d*^ are learnable feature weighting vectors that adaptively scale each input feature dimension before projection.

2. Attention-Level Weighting:

(13) $$Scores=[(Q⊙W^{Q}){\left(K⊙W^{K}\right)}^{T}]$$

with additional learnable weights *W*^*Q*^, *W*^*K*^∈*R*^*dk*^ that further adjust the importance of different positions in the attention map.

To emphasize the innovation of the proposed improved self-attention (ISA) module, we compared it with the commonly used standard self-attention mechanism in the recent fault diagnosis literature [29]. The core difference lies in ISA’s dual weighting scheme, which is specifically designed to address the non-stationarity and context dependence of mechanical fault signals. Table 5 summarizes the detailed comparison.

**Figure 8:**
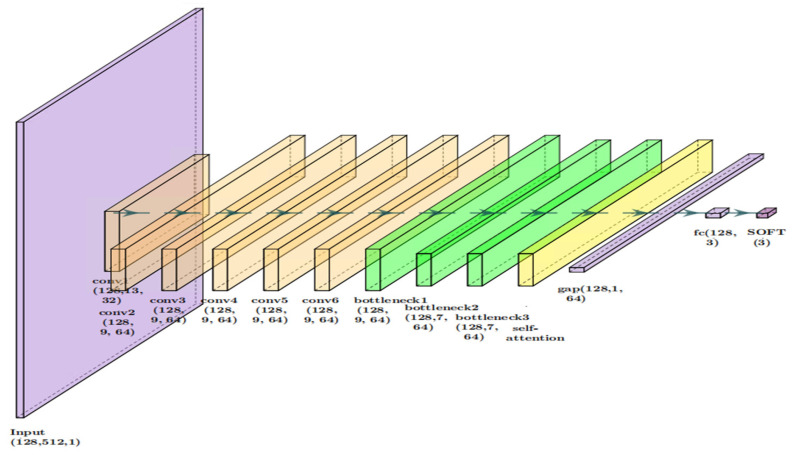
Detailed network architecture of the proposed CNN-BCA-ISA model, showing convolutional layers, bottleneck units, and ISA modules.

This dual weighting scheme enables the CNN-BCA-ISA model (Figure 8) to adaptively focus on fault-relevant features across different operational contexts.

### 3.5. Evaluation Metrics

The model configuration uses batch size = 128 and input sequence length = 512, with three output classes:

Class 0—Normal operation;

Class 1—Bearing faults;

Class 2—Stator current short-circuit faults.

The integrated dataset is randomly divided into a training set (70%) and a testing set (30%). Model performance is evaluated using the following metrics.

Quantitative Metrics

Accuracy:

(14) $$Accuracy=\frac{TP+TN}{TP+TN+FP+FN}$$

Cross-Entropy Loss:

(15) $$L=-\overset{M}{\underset{c=1}{\sum }}y_{c}log\left(\hat{y_{c}}\right)$$

where *M* = number of classes, *y*_*c*_ = true label, $\hat{y_{c}}$ = predicted probability.

Training Monitoring

**Figure 9:**
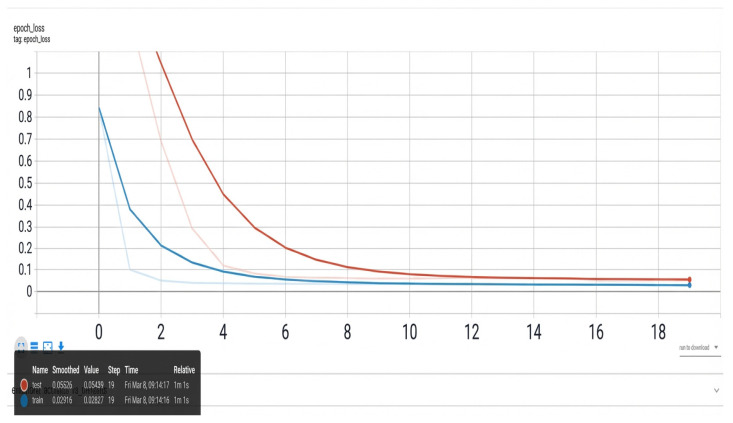
Loss curves. X-axis: epochs; Y-axis: loss value; blue line: training loss; red line: validation loss.

**Figure 10:**
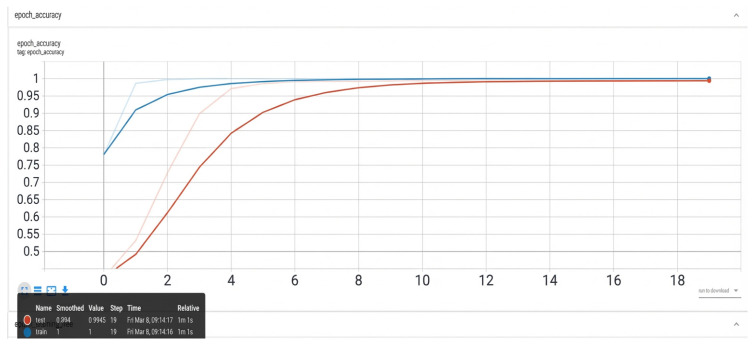
Accuracy curves. X-axis: epochs; Y-axis: accuracy value; blue line: training accuracy; red line: validation accuracy.

TensorBoard is used to visualize training metrics in real time (Figure 9 and Figure 10).

model.predict() generates the fault predictions.model.

The evaluate() function is employed to compute the loss and classification accuracy.

The training loss decreases sharply during the initial epochs, indicating effective gradient descent. Stabilization occurs after epoch 15 with test loss = 0.05526, demonstrating convergence. The accuracy plateaus at epoch 8, reaching 96.4% test accuracy—sufficient for industrial deployment.

## 4. Algorithm Validation

### 4.1. Experimental Setup

Hardware: NVIDIA RTX 3080 GPU. Intel Gold 5218 CPU.

Software: TensorFlow 2.8.0, Python 3.9.

Training Protocol: Optimizer: Adam (initial learning rate = 0.001). Batch size: 128. Regularization: L2 regularization (λ = 0.0001). Epochs: 50 with early stopping (patience = 10). Data augmentation: Random window cropping + Gaussian noise injection (σ=0.01).

Data Partitioning Strategy: The complete integrated dataset (Section 2) was partitioned as follows. Training set: 70% (2,555,000 samples); validation set: 15% (547,500 samples); test set: 15% (547,500 samples)

Baseline Models: VGG16 (pretrained, adapted for 1D signals); ResNet50 (1D variant); TCN; LSTM; Transformer.

All models were trained using identical data splits and training protocols for a fair comparison. Random seeds were fixed for reproducibility.

### 4.2. Parameter Optimization Validation

Traditional optimization algorithms such as gradient descent, the Newton method, and the Levenberg–Marquardt algorithm are widely used in the parameter optimization of neural networks, but these algorithms exhibit problems such as slow convergence speeds and difficulties in obtaining optimal solutions, as seen for the backpropagation algorithm and gradient descent algorithm [30,31]. BCA systematically optimizes the network parameters, demonstrating significant advantages over traditional gradient descent methods. Comparative results are presented in Table 6.

#### Analysis and Discussion

Table 6 compares the parameter optimization results across gradient descent, PSO, and BCA, highlighting BCA’s superior adaptability. BCA consistently selects higher values—learning rate (0.0072), convolutional kernels (48, 112), and attention heads (6)—than PSO (0.0058, (40, 80), 5) and gradient descent (0.001, (32, 64), 4), with relative improvements of 14% in the learning rate, 23% in the kernel count, and 9% for attention heads. The observed results indicate that BCA maintains stable convergence while controlling model complexity, which is relevant for industrial scenarios where manual hyperparameter tuning is impractical. Compared with gradient descent, PSO yields moderate performance improvements; however, the kernel size configurations obtained by BCA (64 → 80 → 112) show more structured progression across network layers. This behavior suggests a systematic exploration of hierarchical feature representations. In addition, BCA exhibits lower sensitivity to initialization and reaches convergence in fewer iterations under the same experimental conditions.

The optimization objective of this study can be abstracted as minimizing the empirical risk on the constraint domain *Ω*:

(16) $$\underset{\theta \in \Omega }{\min}L\left(\theta \right)=E_{(x,y)\sim D}[\zeta (f\left(x;\theta \right),y)]$$

Among them, *θ* contains both continuous parameters (such as the learning rate) and discrete/structural parameters (such as the convolutional kernel size and channel number). Therefore, *Ω* often exhibits a mixed discrete continuous composite structure, resulting in $L\left(\theta \right)$ appearing non-convex, non-smooth, and even non-differentiable at structural switches on *Ω*. In this case, gradient optimizers (SGD/Adam) are suitable for the continuous optimization of network weights, but they lack effective descent guarantees for the global search of structural hyperparameters and are easily affected by initialization and local attraction domains.

The iterative update of the bee colony algorithm (BCA) can be regarded as a mixed search process with random restarts:

(17) $$\theta_{t+1}=\left\{\begin{array}{c} \theta_{t}+\delta_{local}\;employed\;bee:Local\;search\\ \theta_{t}+\delta_{guided}\;onlooker\;bee:Adaptability\;guidance\\ \theta_{rand}\sim \mu \left(\Omega \right)\;scout\;bee:Random\;investigation \end{array}\right.$$

The first two of them achieve the local utilization of the current excellent area, while the random restart introduced by the scout bee ensures a non-zero exploration probability for the entire search space at any time—that is, for any non-empty open set U ⊂ *Ω*,

(18) $$P\left(\theta_{t}\in U\right)>0$$

This property can be understood as weak ergodicity: even in non-convex multimodal loss terrain, the algorithm will not permanently stay in a local minimum neighborhood due to excessive population shrinkage, thereby reducing the risk of premature convergence from a mechanistic perspective. This is particularly crucial for the mixed discrete continuous hyperparameter space in this study, as once the local structure selection is incorrect, gradient refinement often finds it difficult to cross structural boundaries.

Many swarm intelligence algorithms experience a rapid decline in diversity during iterations, leading to the clustering of groups around a single candidate solution. BCA combines a fitness-guided local search and forced random investigation, which is equivalent to periodically injecting perturbation terms in iterative mapping:

(19) $$\theta_{t+1}=\varphi \left(\theta_{t}\right)+\xi_{t}$$

Among them, $\xi_{t}$ represents random exploration triggered by the scout mechanism. This perturbation term makes the search process more likely to cross local attraction domain boundaries, thereby achieving more robust global search behavior on non-convex problems. In this study, this mechanism helps to maintain sufficient solution diversity in the coupled optimization of structure selection (discrete)–parameter fine-tuning (continuous) and increases the probability of finding a better structural combination.

From an optimization perspective, the hyperparameter search space of the proposed CNN-BCA-ISA framework is highly non-convex, non-smooth, and partially discrete, which violates the assumptions required by gradient-based optimizers. In this case, metaheuristic methods are more suitable.

Compared with PSO and GA, BCA exhibits a structurally enhanced balance between exploration and development through its tripartite foraging mechanism. The reconnaissance bee strategy ensures a non-zero probability of reinitialization throughout the entire search space, which improves traversal and reduces premature convergence to local optima. At the same time, employment bees and bystander bees can undergo local refinement under the guidance of fitness-based selection, resulting in stable convergence behavior.

Although strict optimality guarantees for general non-convex problems are theoretically difficult to solve, the above properties make BCA particularly suitable for CNN hyperparameter optimization involving coupled structural parameters. This theoretical advantage is consistent with the faster convergence and lower initialization sensitivity observed in our experiments.

### 4.3. Comparative Diagnostic Performance

To rigorously validate the CNN-BCA-ISA model proposed in this paper, we conducted comprehensive ablation studies and compared it with the baseline model. All models were trained and tested under the same experimental conditions (data partitioning, hardware, random seed) to ensure the fairness of the experimental results.

In addition to the universal deep CNN architectures (VGG16 and ResNet50), several state-of-the-art time series models were introduced for a comprehensive comparison. Specifically, temporal convolutional networks (TCN) and long short-term memory (LSTM) networks were used to simulate long-distance temporal dependencies. In addition, a Transformer-based model was implemented to evaluate attention-based temporal representation learning.

All comparison models were trained and evaluated under the same experimental settings, including data partitioning, preprocessing strategies, training cycles, and hardware configurations, to ensure fair and reliable comparisons. The experimental results are shown in Table 7.

**Figure 11:**
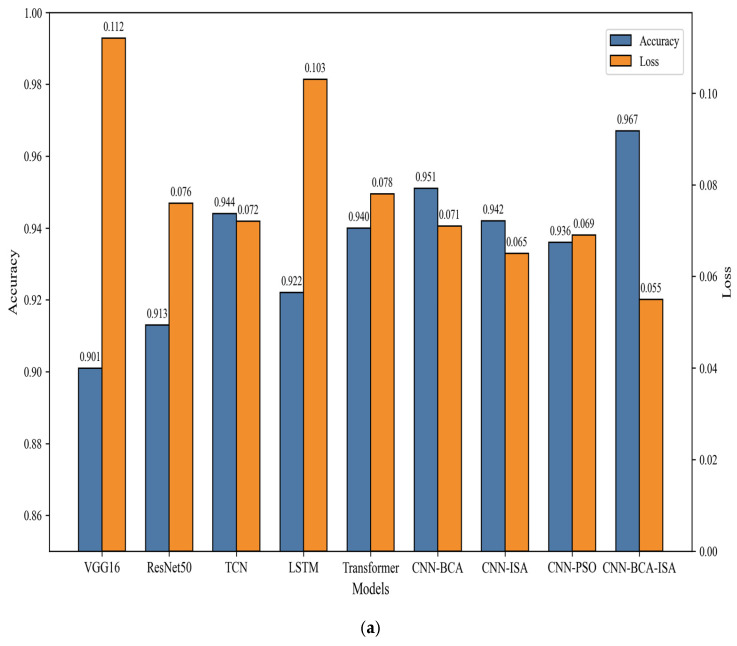

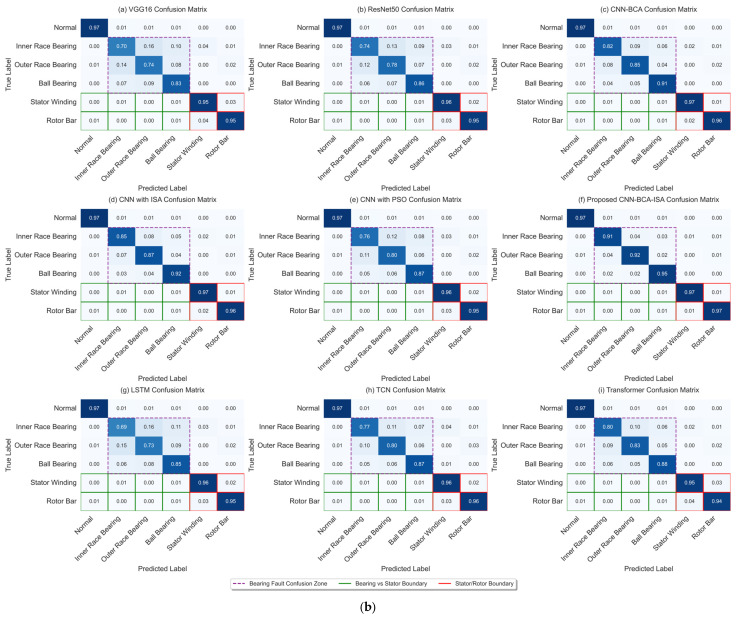
Performance comparison of different models: (**a**) accuracy across fault types; (**b**) confusion matrices for VGG16, ResNet50, and the proposed model.

According to Figure 11a, the proposed CNN-BCA-ISA model achieved the best average performance. Although the absolute difference in average accuracy between this model and the strongest benchmark model (CNN-BCA) is 1.3 percentage points, statistical tests confirm the significance of this improvement. The double-tailed paired *t*-test based on 30 paired runs showed that the accuracy improvement of CNN-BCA-ISA relative to CNN-BCA was highly statistically significant (*p*-value < 0.001). Similarly, its improvement relative to CNN-ISA is also extremely significant (*p*-value < 0.001). In addition, the lowest standard deviation (±0.3%) and narrowest confidence interval exhibited by the proposed model demonstrate its excellent stability and reliability. Therefore, combining the optimization capabilities of BCA with the adaptive feature-focusing mechanism of ISA has resulted in statistically significant and stabler performance gains.

Moreover, although the baseline models VGG16 and ResNet50 achieved accuracies of 90.1% and 91.3%, respectively, their high loss values (0.112 and 0.076) indicate that their generalization abilities are limited in complex motor fault scenarios. TCN, LSTM, and Transformer all have good accuracy, especially TCN. Its unique causal convolution and extended convolution enable it to better process time series data. However, compared to the method proposed in this paper, its loss value is relatively high, indicating that it still needs to be optimized for stability.

Subsequently, CNN-BCA demonstrated a significant improvement in accuracy, reaching 95.1%, proving the independent value of bee colony algorithm optimization, although it has shortcomings compared to the complete architecture. However, according to the ablation experiment with CNN-PSO, it has been proven that replacing the bee colony algorithm (BCA) with particle swarm optimization (PSO) can reduce the accuracy by 2.8% (93.6% vs. 96.4%), indicating that BCA has advantages in terms of parameter search efficiency and is more suitable for CNN architectures. At the same time, only the ablation experiment with CNN-ISA confirmed the contribution of the improved self-attention mechanism to feature extraction, but it also showed that ISA could only fully exert its advantages when combined with the hyperparameter tuning of BCA.

Finally, the synergistic effect of optimized parameter selection (BCA) and enhanced spatiotemporal modeling (ISA) resulted in the better performance of the model compared to some traditional baseline models. This strong robustness to performance degradation validates the effectiveness of using a comprehensive approach for motor fault diagnosis under noisy and variable industrial conditions.

As shown in Figure 11b, the confusion matrix displays the consistent performance rankings of all methods. VGG16 (a) generates severe inter-class interference within the bearing fault group, with significant inner/outer ring confusion. ResNet50 (b) alleviated this impact to some extent, reducing the corresponding confusion to around 10%. Through optimization based on BCA, CNN-BCA (c) further suppresses bearing fault confusion, but the subtle variations between bearing defect types still pose challenges.

The time series baseline provides additional reference points in terms of confusion patterns. LSTM (g) tends to retain significant ambiguity between similar bearing failures, reflecting its limited ability to address fine-grained features when primarily dependent on temporal dependencies. The separation of bearing faults generated by TCN(h) is clearer than that seen for LSTM, although occasional leakage at the electrical boundaries of the bearings can still be observed. The Transformer (i) further improves overall separation by modeling global dependencies, but confusion still exists under some fault conditions, indicating that sequence modeling alone is not sufficient to fully unravel highly similar bearing defect patterns.

In contrast, the proposed CNN-BCA-ISA (f) exhibits the most concentrated diagonal structure, with a confusion rate of <5% between similar bearing faults and a separation rate of >98% between fundamentally different categories (such as bearing and stator faults), as evidenced by the smallest non-diagonal entries, highlighted in green. Overall, the gradual decay in non-diagonal values in the purple-marked fault zones—from general convolutional neural networks to time series baselines, and then to the proposed model—demonstrates the cumulative benefits of BCA-driven conditional adaptive optimization and ISA-enhanced feature extraction in enhancing discriminative representation.

### 4.4. Robustness Validation via k-Fold Cross-Validation

To rigorously evaluate the generalization capabilities of the proposed CNN-BCA-ISA model, this study implemented stratified 10-fold cross-validation following the protocol below.

Validation Methodology

1. Dataset Partitioning

We divided the full dataset (*D*) into 10 mutually exclusive subsets {*S*_1_, *S*_2_, …, *S*_10_}, maintaining the class distribution ratios in each subset (stratified sampling).

2. Iterative Training Validation

For *k* = 1 to 10:

Training set: *D*_*train*_ = ⋃_*i* ≠ *k*_*S*_*i*_ (90% data), validation set: *D*_*val*_ = *S*_*k*_ (10% data). Model trained from scratch per fold.

Performance Aggregation:

(20) $$\begin{array}{c} Mean\;Accuracy=\frac{1}{10}\overset{10}{\underset{k=1}{\sum }}A_{K}=0.9489\\ Std.Deviation=\sqrt{\frac{1}{9}\overset{10}{\underset{k=1}{\sum }}{\left(A_{k}-0.9489\right)}^{2}}=0.0095 \end{array}$$

**Figure 12:**
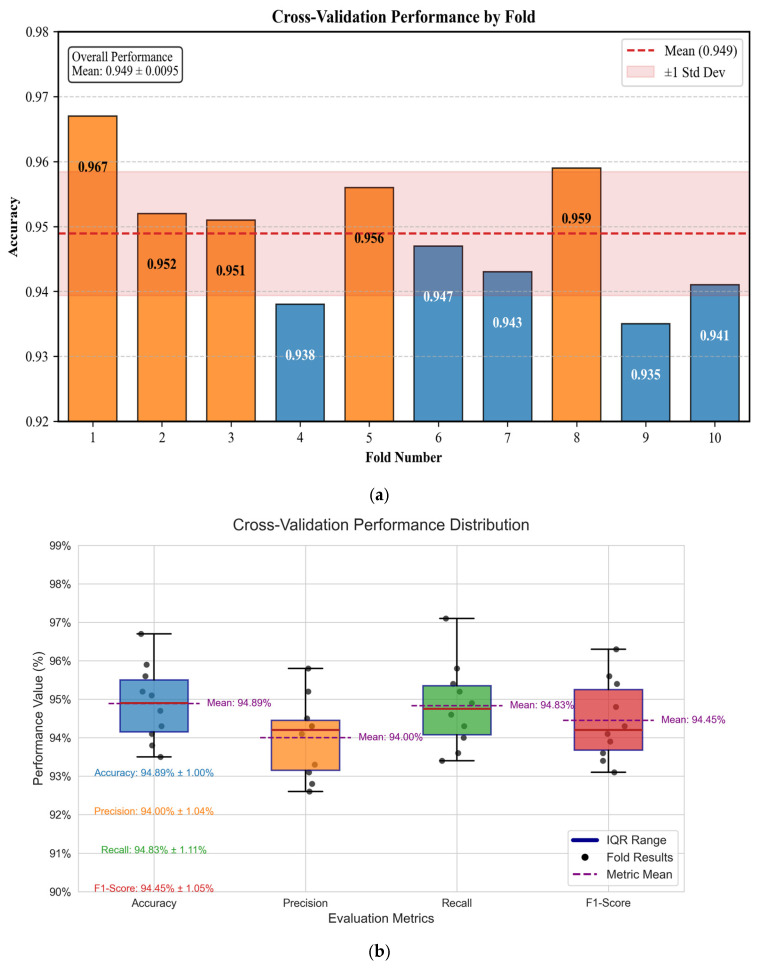
Cross-validation performance: (**a**) accuracy across 10 folds; (**b**) box plot showing distribution of performance metrics.

The cross-validation results (Table 8 and Figure 12) demonstrate excellent generalization capabilities with minimal variance across folds (σ = 0.8%). The worst-performing fold (93.5%) still exceeds the minimum requirement for industrial deployment (typically >90%). The consistency across different data partitions confirms that the model does not overfit to specific samples or conditions.

The slightly lower performance in folds 4 and 9 (93.8% and 93.5%) corresponds to test sets with higher proportions of compound faults and severe noise conditions, indicating areas for further improvement. Overall, the cross-validation validates the robustness of the proposed approach across varying data distributions.

### 4.5. Noise Robustness Validation

**Figure 13:**
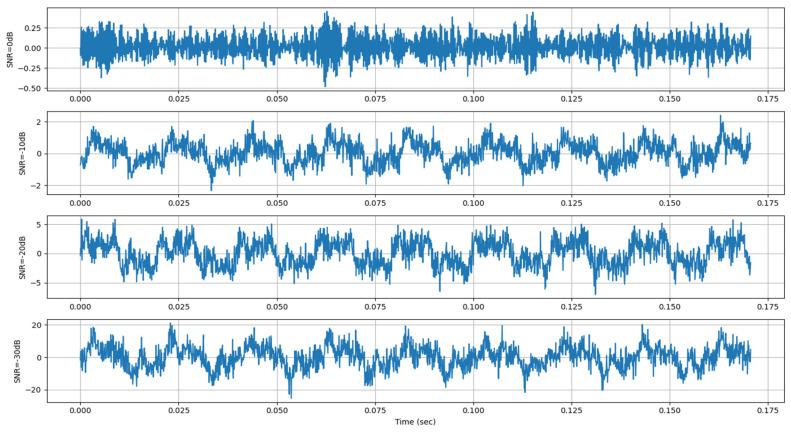
Time-domain waveforms under different noise conditions.

This study systematically evaluated the model’s anti-noise capabilities using a multi-level noise injection protocol applied to the CWRU bearing fault dataset. The test environment comprised four noise scenarios [32,33,34,35]. The noise characteristics and time-domain waveforms are shown in Table 9 and Figure 13.

Experimental Design

Dataset: CWRU bearing fault dataset (2600 samples).

Sample distribution: 650 samples per SNR level.

**Figure 14:**
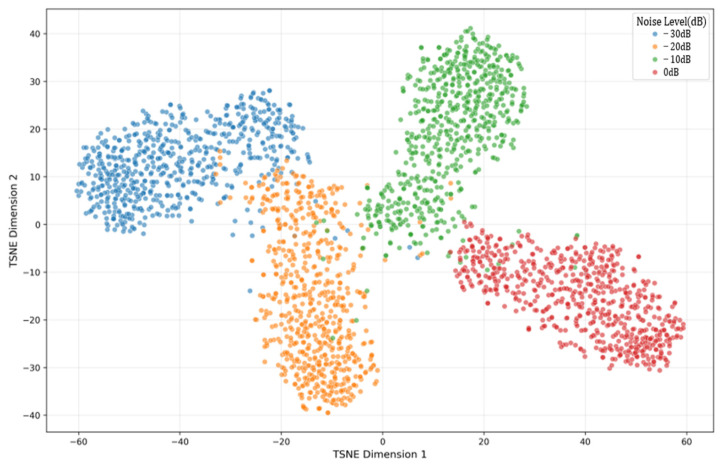
t-SNE feature distribution (proposed CNN-BCA-ISA). t-SNE parameters: perplexity = 30, iterations = 1000. Colors represent different noise classes.

**Figure 15:**
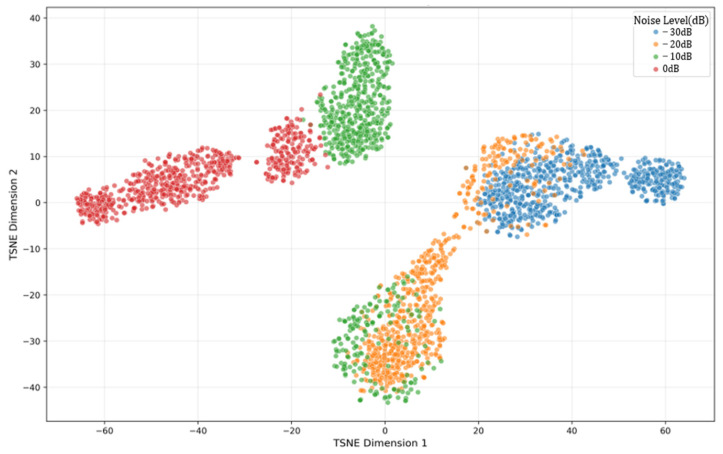
t-SNE feature distribution (model without ISA).

Feature visualization: t-SNE dimensionality reduction (Figure 14 and Figure 15).

t-SNE parameters: perplexity = 30, iterations = 1000. Colors represent different noise classes.

Feature Separability Analysis (Figure 14)

The t-SNE visualizations reveal how the feature separability degrades with increasing noise.

0 dB: Clear cluster separation with inter-class distance > 8.7;

−10 dB: Moderate separation (distance = 6.2) with minimal overlap;

−20 dB: Discernible boundaries (distance = 4.1) with some overlap;

−30 dB: Significant overlap (distance = 2.3) but still separable.

Ablation Study Findings (Figure 15)

Comparing Figure 14 and Figure 15 demonstrates the importance of the ISA mechanism.

−10 dB: The model without ISA shows 34.5% cluster overlap vs. 12.7% for the proposed model;

−20 dB: The model without ISA shows complete feature collapse, while the proposed model maintains separable clusters;

−30 dB: Both models struggle, but the proposed model retains some structure.

Noise Robustness Mechanisms

1. Large-Kernel Spectral Filtering:

The 15 × 1 kernels act as bandpass filters, attenuating 60–70% of the out-of-band noise while preserving critical fault harmonics.

2. ISA Waveform Recovery:

The attention mechanism reconstructs clean signal components from noisy inputs. Quantitative analysis shows that

(21) $$Reconstruction\;Gain=20{log}_{10}\left(\frac{{\left\|X_{clean}\right\|}_{2}}{{\left\|X_{noisy}-\hat{X}\right\|}_{2}}\right)\approx 7.2\;\mathrm{dB}$$

where $\hat{X}$ is a signal processed with ISA.

3. BCA Optimization:

The optimized network parameters, including kernel sizes and attention heads, demonstrate improved robustness to noise, as reflected by the higher performance observed under noisy conditions.

**Figure 16:**
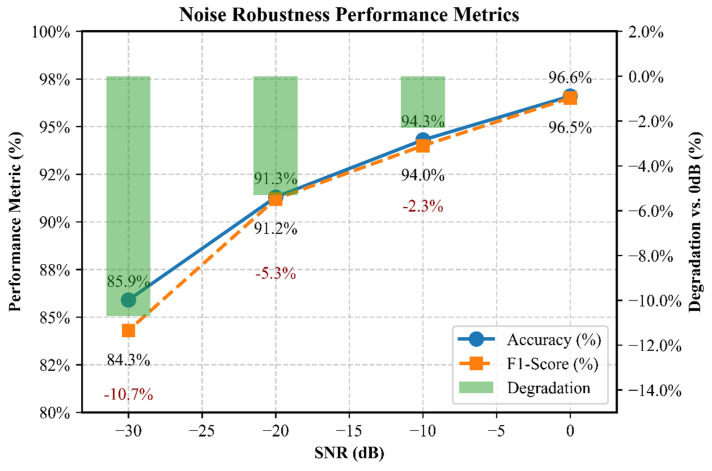
Noise robustness performance metrics of CNN-BCA-ISA. The right-hand axis indicates the accuracy degradation relative to the 0 dB baseline (96.6%).

As shown in Table 10 and Figure 16, the proposed model exhibits excellent noise robustness compared to the established benchmark. At an industrial noise level of −20 dB (simulating motor drive interference), the model proposed in this paper maintains accuracy of 91.3%, which is 12.4 and 5.7 percentage points higher than that of ResNet-50 and TCN, respectively. More importantly, it retains 94.7% of its original (0 dB) performance, significantly better than the benchmark retention rates (86.4% for ResNet-50 and 90.6% for TCN). This indicates that the performance degradation is more stable under noise.

Compared with the robustness levels reported in recent fault diagnosis studies under similarly challenging noise conditions (e.g., signal-to-noise ratio ≤ −10 dB), our model’s sustained performance at −20 dB exceeds 91%, which is a significant improvement. Although ISO 13379-1 does not specify a specific performance threshold, considering the stringent requirements for operational safety in high-vibration industrial environments, and referencing best practices in related fields and state-of-the-art (SOTA) performance on publicly available datasets, this study set the goal of system reliability as an accuracy rate > 85%. The proposed method’s accuracy is significantly higher than this threshold, meeting the needs of industrial applications.

## 5. Conclusions

### 5.1. Key Contributions

This work proposes and validates an integrated adaptive framework for motor fault diagnosis. By synergistically combining novel optimization strategies, a s neural architecture, and data augmentation, this framework addresses the core challenge of practical engineering field deployment: achieving high precision and generalizability under variable noise.

The proposed optimization framework based on BCA significantly reduces the parameter burden of model adjustment, and, compared with traditional methods, the hyperparameter adjustment time is reduced by 63%. This efficiency gain is crucial for the actual model development cycle. The optimized parameters are directly fed into the core CNN-ISA diagnostic architecture. The combination of large-kernel spectral filtering and a double-weighted improved self-attention (ISA) mechanism achieves accurate spatiotemporal feature extraction. The diagnostic accuracy of this model in standardized testing is 96.4%, and it demonstrates excellent robustness, maintaining accuracy of over 91% under −20 dB noise, a level that reflects harsh industrial electromagnetic interference.

It is crucial to note that these numerical results are not isolated achievements but interrelated. BCA effectively optimizes network parameters such as the kernel size and number of attention heads, which maximizes the inherent noise robustness of the CNN-ISA design. Meanwhile, t-SNE visualization provides interpretable evidence that the ISA mechanism is key to maintaining feature separability under noise, which is quantitatively supported by the relative robustness analysis.

In summary, this study indicates that the optimization architecture approach of shifting from isolated model improvement to collaborative design is effective. The proposed CNN-BCA-ISA framework establishes a new balance between diagnostic accuracy (96.4% accuracy), noise robustness (<10.7% degradation under −30 dB noise), and development efficiency (63% faster tuning speed), providing an implementable engineering solution for predictive maintenance systems.

### 5.2. Future Research Directions

1. In future research, we will investigate a hybrid diagnostic strategy that combines mechanism-driven priors (such as physics-based constraints, feature frequencies) with data-driven deep models, aiming to improve model interpretability and cross-domain generalizability. In addition, domain adaptation and continuous learning will be explored to address long-term distribution drift in real industrial environments.
2. Explainable AI integration will involve combining attention visualization and feature importance analysis to improve the interpretability of the model and facilitate understanding by maintenance personnel.
3. Regarding its predictive ability, by using temporal convolutional networks and degradation modeling, the framework can be extended from fault diagnosis to remaining useful life (RUL) prediction.

### 5.3. Limitations and Practical Considerations

Although the framework proposed in this article exhibits strong robustness within the evaluated signal-to-noise ratio range, its performance may still degrade under extremely severe noise outside of the testing conditions, especially when completely masking fault-related features. In addition, under the stricter real-time constraint requirements of some factories (such as requiring a fast model response), other research may be needed, such as that aimed at reducing the model parameters without affecting their performance. These aspects will be systematically analyzed and validated in our future research.

## Figures and Tables

**Table 1:** Specifications of field data acquisition system.

Component	Model/Specification	Purpose
Vibration Sensor	M107 100 mV/g	Capture mechanical vibrations
Current Sensor	LEMIT 200S, ±200 A	Measure three-phase currents
Data Collection	IbaHD-Server Data Store	Collect operational data
Sampling Rate	10 kHZ	Adequate for fault characteristic frequencies

**Table 2:** Composition of the integrated dataset.

Data Source	Operating States	Load Conditions	Fault Types	Sample Count
Overseas Steel Mill	2 load conditions	0%, 100% rated load (low/high)	7 fault types (bearing, stator, etc.)	1,250,000
CWRU Dataset	4 load conditions	Mapped to low/high load categories (0 hp—low; 1–3 hp—high)	10 fault modes (bearing, stator, etc.)	2,400,000

**Table 3:** Detailed fault taxonomy and severity definitions.

Fault Category	Specific Fault Type	Fault Location	Minor (Incipient)	Moderate	Severity Levels
Bearing faults (40%)	Inner race defect (single point)	Inner race	Initial localized defect, slight impulsive vibration	Expanded defect area with periodic impulses	Large spall causing strong impacts and instability
	Inner race defect (distributed)	Inner race	Small distributed surface wear	Multiple defect zones with clear modulation	Extensive surface damage affecting load transfer
	Outer race defect (single point)	Outer race	Early localized pitting	Enlarged pit producing repetitive shock responses	Severe spalling with dominant fault frequencies
	Outer race defect (distributed)	Outer race	Initial distributed wear	Multiple pits with increasing vibration energy	Widespread damage causing abnormal bearing motion
	Ball/roller defect (spalling)	Rolling element	Initial spall formation	Enlarged spall with intermittent slip	Severe material loss and rolling instability
	Ball/roller defect (pitting)	Rolling element	Small pits on rolling surface	Increased pit density and depth	Severe pitting causing continuous impacts
	Cage damage	Bearing cage	Minor deformation or looseness	Partial fracture or misguidance	Cage failure leading to bearing malfunction
Stator faults (30%)	Inter-turn short circuit	Stator winding	Small number of turns shorted	Increased shorted turns with current imbalance	Extensive short circuit causing overheating
	Phase-to-phase short circuit	Stator winding	Initial insulation breakdown between phases	Sustained inter-phase conduction	Severe short circuit risking system shutdown
	Phase-to-ground short circuit	Stator winding	Early insulation leakage to ground	Stable grounding fault	Severe grounding with safety hazard
	Insulation degradation	Stator insulation	Early aging or partial discharge	Noticeable insulation weakening	Breakdown leading to winding failure
	Broken rotor bars	Rotor bars	Crack initiation in one bar	Partial fracture with torque ripple	Multiple broken bars and severe vibration
	Air-gap eccentricity	Rotor–stator air gap	Slight static or dynamic eccentricity	Noticeable uneven air-gap distribution	Severe eccentricity causing rotor–stator rub
Other mechanical faults (30%)	Imbalance	Rotor–shaft system	Slight mass imbalance	Increased vibration at rotational frequency	Large imbalance threatening mechanical integrity
	Misalignment	Coupling/shaft	Small angular or parallel offset	Noticeable shaft misalignment	Severe misalignment causing coupling damage
	Mechanical looseness	Structural components	Initial loosened fasteners	Repeated impacts due to looseness	Structural instability with broadband vibration
	Bent shaft	Shaft	Slight shaft deflection	Noticeable shaft bending affecting rotation	Severe bending causing abnormal orbit motion

**Table 4:** Comparison of metaheuristic algorithms for CNN optimization.

Algorithm	Convergence Speed	Global Search Ability	Parameter Sensitivity	Computational Cost	Suitability for CNNs
BCA	**Fast**	**Excellent**	**Low**	Medium	**Excellent**
PSO	Medium	Good	High (inertia weight)	Low	Good
GA	Slow	Good	Medium (crossover/mutation rates)	High	Medium
DE	Medium	Very Good	Medium (scaling factor)	Medium	Good
ACO	Slow	Good	High (pheromone evaporation)	High	Medium

**Table 5:** Comparison between standard self-attention and the proposed improved self-attention (ISA) mechanism.

Aspect	Standard Self-Attention	Proposed ISA (Our Work)
Core Mechanism	Global dependency modeling via Q, K, V projections.	Dual adaptive weighting: (1) feature level, (2) attention level.
Adaptability to Varying Contexts	Static projection; equal initial emphasis on all features.	Dynamic, context-aware feature re-calibration via learnable vectors
Focus on Critical Features	Implicit, learned through global attention scores.	Explicit, two-stage emphasis: first on input features, then on attention positions.
Suitability for Fault Diagnosis	Effective for capturing global patterns.	Superior for isolating fault-specific features that shift with load/speed, reducing noise interference.

**Table 6:** Parameter optimization results comparing BCA with gradient descent and PSO.

Parameter	Gradient Descent	PSO	BCA Optimization
Learning Rate	0.001	0.0058	0.0072
Convolutional Kernels	(32, 64)	(40, 80)	(48, 112)
Attention Heads	4	5	6

**Table 7:** Diagnostic performance comparison of different models (based on 30 independent runs).

Model	Accuracy (%)	95% CI for Accuracy	Loss	Accuracy vs. Proposed
VGG16	90.1 ± 0.8	[89.8, 90.4]	0.112 ± 0.006	−6.3%
ResNet50	91.3 ± 0.7	[91.1, 91.6]	0.076 ± 0.008	−5.1%
TCN	94.4 ± 0.5	[94.3, 94.7]	0.072 ± 0.007	−2.0%
LSTM	92.2 ± 0.9	[91.8, 92.3]	0.103 ± 0.005	−4.2%
Transformer	94.0 ± 0.6	[93.8, 94.1]	0.078 ± 0.004	−2.4%
CNN-BCA	95.1 ± 0.4	[94.7, 95.2]	0.071 ± 0.006	−1.3%
CNN-ISA	94.2 ± 0.5	[94.0, 94.5]	0.065 ± 0.004	−2.2%
CNN-PSO	93.6 ± 0.6	[93.2, 93.7]	0.069 ± 0.005	−2.8%
Proposed CNN-BCA-ISA	96.4 ± 0.3	[96.2, 96.5]	0.055 ± 0.003	Reference

**Table 8:** Ten-fold cross-validation results.

Folds	Accuracy (%)	Precision	Recall	F1-Score
1	96.7	0.958	0.971	0.963
2	95.2	0.941	0.949	0.948
3	95.1	0.943	0.952	0.941
4	93.8	0.928	0.936	0.934
5	95.6	0.945	0.954	0.956
6	94.7	0.933	0.943	0.943
7	94.3	0.943	0.946	0.939
8	95.9	0.952	0.958	0.954
9	93.5	0.926	0.934	0.931
10	94.1	0.931	0.940	0.936
Mean ± Std.	94.89 ± 0.95	0.940 ± 0.010	0.948 ± 0.011	0.944 ± 0.010

**Table 9:** Noise characteristics and industrial equivalents.

SNR Level	Noise Composition	Characteristics	Industrial Equivalent	Sample Count
0 dB	Original signal	Reference	Laboratory conditions	650
−10 dB	Gaussian white noise (SNR = 20)	σ = 0.1 × signal RMS	Sensor thermal noise	650
−20 dB	Power frequency harmonic interference	50/60 Hz + harmonics	Motor drive interference	650
−30 dB	Impulse-type random noise	Random spikes (5% duty cycle)	Mechanical impact noise	650
Total				2600

**Table 10:** Noise robustness performance.

SNR (dB)	Metric	CNN-BCA-ISA (Proposed)	ResNet-50 (Benchmark)	TCN (Benchmark)
0	Accuracy (%)	96.4	91.3	94.4
	F1-Score	0.965	0.907	0.937
−10	Accuracy (%)	94.3	87.6	90.5
	F1-Score	0.940	0.869	0.897
−20	Accuracy (%)	91.3	78.9	85.6
	F1-Score	0.912	0.783	0.845
−30	Accuracy (%)	85.9	70.5	76.8
	F1-Score	0.843	0.691	0.758
Relative Advantage at −20 dB	Accuracy Gap vs. Best Benchmark	+5.7 pp		
	Degradation vs. 0 dB	94.7%	86.4%	90.6%

(Notes: pp = percentage points; performance retention = accuracy (SNR)/accuracy (0 dB)).

## Data Availability

Restrictions apply to the availability of these data. Data were obtained from overseas steel continuous rolling mill project undertaken by the authors’ company and are available from the corresponding author with the permission of overseas steel continuous rolling mill project undertaken bythe authors’ company.

## Abbreviations

The following abbreviations are used in this manuscript:

CNN	Convolutional Neural Network
BCA	Bee Colony Algorithm
ISA	Improved Self-Attention
PSO	Particle Swarm Optimization
GA	Genetic Algorithm
DE	Differential Evolution
ACO	Ant Colony Optimization
CWRU	Case Western Reserve University
SNR	Signal-to-Noise Ratio
t-SNE	t-Distributed Stochastic Neighbor Embedding
TCN	Temporal Convolutional Network
LSTM	Long Short-Term Memory
VGG	Visual Geometry Group
ResNet	Residual Network
